# Man of Steel Syndrome: Silicone and Mineral Oil Injections With Associated Hypercalcemia, Hypophosphatemia, and Proximal Muscle Weakness

**DOI:** 10.1002/jbm4.10208

**Published:** 2019-07-29

**Authors:** Arash Shirvani, Nadine E Palermo, Michael F Holick

**Affiliations:** ^1^ Section of Endocrinology, Diabetes & Nutrition, Department of Medicine, Vitamin D, Skin, and Bone Research Laboratory Boston University School of Medicine (BUSM) Boston MA USA; ^2^ Division of Endocrinology Diabetes and Hypertension, Brigham and Women's Hospital, Harvard Medical School Boston MA USA

**Keywords:** PTH/ VITAMIN D /FGF‐23, SILICONE/GRANULOMA, HYPERCALCEMIA, HYPOPHOPHATEMIA, 1,25‐DIHYDROXYVITAMIN‐D

## Abstract

Silicone/mineral oil‐induced granulomas have been described as an inflammatory granulomatous response when silicone/mineral oil is injected for cosmetic purposes. These sclerosing granulomas can lead to hypercalcemia. Here we present a 33‐year‐old man with hypercalcemia, hypophosphatemia, progressively worsening fatigue, severe proximal muscle weakness, and depression. He had an athletic build with increased muscle bulk and several areas of indurated, nontender, firm, well‐circumscribed lesions in the subcutaneous tissue of his anterior pectoralis, triceps, and biceps bilaterally because of injecting himself with silicone/mineral oil‐based product into his muscles. Sclerosing granulomas were diagnosed on the MRI. He had extremely low or undetectable serum levels of 25‐hydroxyvitamin D [25(OH)D], and persistently elevated serum levels of 1,25‐dihydroxyvitamin D [1,25(OH)_2_D] and calcium. He developed hypophosphatemia associated with elevated levels of fibroblast growth factor 23 (FGF‐23) and severe proximal muscle weakness. Treatment with systemic steroids, furosemide, calcitonin, ketoconazole, and denosumab resulted in a significant decrease in his serum calcium, but with minimal impact on his hypophosphatemia and fatigue.Correcting his severe vitamin D deficiency with small doses of vitamin D and raising his blood level of 25(OH)D from undetectable to 10 ng/mL without significantly affecting his serum calcium or phosphate was effective in reversing his severe proximal muscle weakness, permitting him to lift his head and to be free of his wheelchair. Although measurement of the 1,25(OH)_2_D level is not mandatory in all cases of hypercalcemia, it is indicated in a patient who has low serum PTH levels. Clinicians need to be aware that vitamin D deficiency can cause severe proximal muscle weakness such that the patient is unable to lift his head from his chest or ambulate. This may lead to a psychiatric disorder misdiagnosis. © 2019 The Authors. *JBMR Plus* is published by Wiley Periodicals, Inc. on behalf of the American Society for Bone and Mineral Research.

## Introduction

Silicone/mineral oil injected for cosmetic purposes can stimulate an inflammatory granulomatous response. The sclerosing granulomas resulting from mineral oil or silicone injections have been associated with severe and recurrent hypercalcemia.^1^, ^2^, ^3^ The mechanism for the silicone/mineral oil granuloma‐induced hypercalcemia has been demonstrated to be based on increased serum 1,25‐dihydroxyvitamin D [1,25(OH)_2_D] because of macrophage synthesis of and release of 1,25(OH)_2_D.^1^, ^4^ We describe a case of severe recurrent hypercalcemia and hypophosphatemia associated with extrarenal production of 1,25(OH)_2_D from sclerosing granulomas after injections of silicone/mineral oil for cosmetic reasons.

## Clinical Vignette

A 33‐year‐old white male presented to our endocrine clinic with an 11‐month history of progressively worsening fatigue, nausea, and polyuria. He had been hospitalized approximately 10 times for severe hypercalcemia with hypophosphatemia.

His past medical history was significant for depression, bipolar disorder, seizure disorder with change for personality, and a benign thyroid nodule. In recent months, he expressed suicidal ideation and was undergoing active treatment for depression. He had no history of fractures, but had several small kidney stones. He was not currently taking any vitamins, supplements, or antacids. He had no current use or previous history of tobacco or alcohol abuse. He had no pets and reported no exposure to other animals.

He denied weight loss, fever, chills, cough, or recent travel. He lived at home with his wife and two children. Prior to hospitalizations for hypercalcemia, he was very active, an avid runner, and involved in competitive weightlifting. His chronic medications included venlafaxine, omeprazole, divalproex sodium, and dexamphetamine. He had been on prednisone for the last 6 months and was started on alendronate approximately 3 weeks prior to this clinic visit for his persistent hypercalcemia. He admitted to injecting himself with silicone/mineral oil‐based product that he purchased on the Internet (the product was advertised as a nonandrogen steroid that could improve muscle size) into his pectoralis majors, biceps, and triceps to enlarge these muscles volumetrically during bodybuilding. Family history was notable for nephrolithiasis in his mother. His mother did not have hypercalciuria. He had no known family history of calcium, bone, thyroid, or other endocrinopathies.

**Figure 1:**
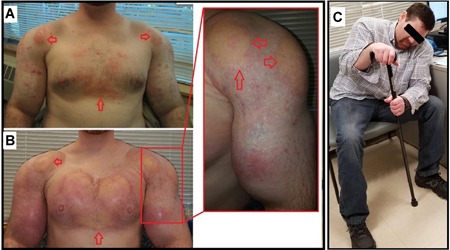
Severe proximal muscle weakness in silicone/mineral oil‐induced granulomas. The male patient had impressive man‐of‐steel‐like firm muscle bulk and several indurated, nontender firm areas in the subcutaneous tissue of his anterior pectoralis major, triceps, and biceps as well as inflammatory acne over his anterior chest (red arrows) in the first visit (*A*) and after 4 years (*B*). Patient presented with severe fatigue that progressed to severe proximal muscle weakness, resulting in him being unable to lift his head or stand from a sitting position without assistance (*C*). His extreme fatigue and severe proximal muscle weakness markedly improved so that he was able to ambulate after receiving small doses of vitamin D_3_ (400 IUs daily) and raising his blood level of 25(OH)D from <4 ng/mL (lowest detection limit for the assay) to 10 ng/mL.

On physical examination, he was an ambulatory male with an athletic build in no apparent distress. He was mentally oriented ×3. He oriented as to who he is, where he is, and when it is. He had increased muscle bulk that was rock hard and several areas of firm, indurated, nontender areas in the subcutaneous tissue of his anterior pectoralis, triceps, and biceps bilaterally, as well as inflammatory acne over his anterior chest (Fig. 1 *A* and *B*). He did not complain of bone pain, and on physical examination he had no periosteal tenderness of his sternum, anterior tibia radius, or ulna. Cardiopulmonary, abdominal, neurologic, and lymph node examinations were normal.

His laboratory testing revealed his serum calcium level was 15.2 mg/dL (normal range 8.4 to 10.2 mg/dL), his ionized calcium level was >7.8 mg/dL, and his phosphorus level was 2.3 mg/dL (normal range 2.7 to 4.5 mg/dL). Several months previously his serum calcium level had exceeded 18 mg/dL and his phosphorus level had been 0.9 mg/dL. His levels of hemoglobin, glucose, albumin, total protein, chloride, potassium, sodium, magnesium, bilirubin, liver function tests, and alkaline phosphatase, as well as blood cell and platelet counts were normal. The 24‐hour urine collection period was preceded by 3 days with the patient on a hypophosphoric diet. The urine calcium level was 17.3 mg/dL, the urine phosphorus was 31.1 mg/dL, and the urine creatinine was 675 mg/dL. The calculated tubular reabsorption of phosphate (TRP) was 0.97 (normal range 0.85 to 1.00) and the tubular threshold maximum for phosphorus per glomerular filtration rate (TMP/GFR) was 1.36 mmol/L (normal range 2.5 to 4.2 mg/dL). His PTH level was 11 pg/mL (normal range 15 to 90 pg/mL), and the PTHrP was 22 pg/mL (normal range 15 to 65 pg/mL), and the thyroid‐stimulating hormone level was 1.9 mIU/L (normal range 0.3 to 5.0 mIU/L). The creatinine level was 2.25 mg/dL, and the GFR was 34 mm^3^/min/1.73 m^3^. Additional biochemical evaluation was notable for 25(OH)D of 17 ng/mL (normal range 20 to 100 ng/mL) and 1,25(OH)_2_D of 101 pg/mL (normal range 15 to 80 pg/mL); his levels of serum protein electrophoresis (SPEP) and urine protein electrophoresis (UPEP) were normal.

**Figure 2:**
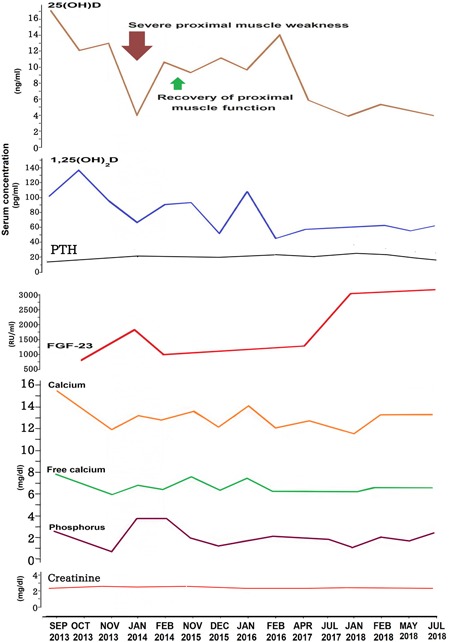
Laboratory results. The elevated serum levels of 1,25‐dihydroxyvitamin D [1,25(OH)_2_D] with a low serum level of 25‐hydroxyvitamin D [25(OH)D] and suppressed PTH were consistent with ectopic production of 1,25(OH)_2_D. His extreme fatigue and proximal muscle weakness were associated with undetectable serum levels of 25(OH)D (red arrow). Treatment with 400 IUs of vitamin D3 daily raised his blood level of 25(OH)D from undetectable (<4 ng/mL) to 10 ng/mL. This resulted in a dramatic improvement in his proximal muscle function and fatigue (green arrow).

Over subsequent months he received denosumab, prednisone, ketoconazole, furosemide, calcitonin, and hydration, but continued to have a persistently elevated serum calcium level (corrected calcium 12.2 mg/dL), an ionized calcium level of 7 mg/dL, a normal phosphorus level (3.2 mg/dL), with a creatinine level of 2.3 mg/dL. The GFR was 32 mm^3^/min/1.73 m^3^, and urea nitrogen was 33 mg/dL. The serum levels of albumin, total protein, chloride, potassium, sodium, magnesium, bilirubin, alanine aminotransferase, aspartate aminotransferase, osteocalcin, and alkaline phosphatase, as well as the random urine N‐telopeptide level were normal. A follow‐up PTH level was 9 pg/mL (normal range 11 to 90 pg/mL); 25(OH)D was <4 ng/mL (detection limit for the assay); 1,25(OH)_2_D was 35 pg/mL. FGF‐23 levels were markedly elevated with values ranging from 665 to 3227 RU/mL (normal <180 RU/mL). The results of his lab tests are shown in Fig. 2. Four months later he presented with a new symptom of extreme fatigue. He was unable to lift his head from his chest, to ambulate, or to get from a sitting to standing position without the assistance of a cane (Fig. 1 *C*). An initial evaluation by a psychiatric consult at an outside hospital concluded that this was caused by his depression and was intentional. Further psychiatric evaluation was recommended.

**Figure 3:**
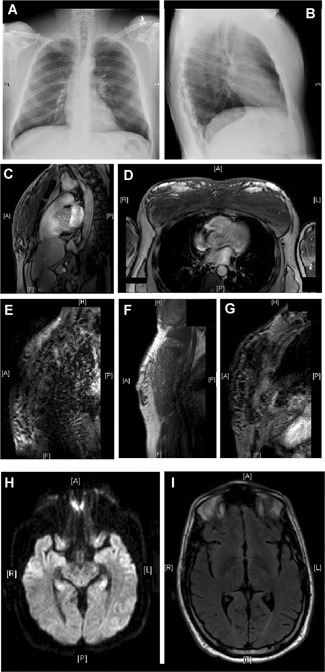
Radiologic findings in the patient. Chest radiography showed multiple peripheral ground glass opacities scattered throughout the visualized portions of the lungs. There were tiny scattered subcentimeter punctate hyperdensities seen within the lung bases that possibly represent calcified granulomas (panel *A* and *B*). MRI of the chest revealed enlargement and heterogeneity with scattered foci of hyperintensity through the sternal head of the pectoralis major and visualized deltoid and bicep brachii muscles bilaterally and marked dermal thickening and heterogeneity of the subcutaneous tissue of the anterior chest wall (*C*, *D*). MRI of his upper extremity showed subcutaneous fatty stranding of the visualized portions of the upper extremities with multiple associated punctate calcifications. There is marked heterogeneity and numerous foci of fluid attenuation and fat throughout the majority of the musculature of the upper extremities, particularly involving the biceps, triceps, and deltoids (*E–G*). MRI of his brain did not detect any mass, hemorrhage, cortical dysplasia or heterotopia, recent infarct, or enhancing parenchymal lesion. No abnormal meningeal enhancement noted. A 1.2‐cm area isointense to CSF related to the left transverse sinus may be a prominent arachnoid granulation. Very mild nonenhancing white matter hyper‐intensities had been reported (*H, I*).

Chest radiography showed multiple peripheral ground glass opacities scattered throughout the visualized portions of the lungs (Fig. 3 *A* and *B*). CT of his upper extremity and chest showed subcutaneous fatty stranding of the visualized portions of the upper extremities and anterior chest wall with multiple associated punctate calcifications. There is marked heterogeneity and numerous foci of fluid attenuation and fat throughout the majority of the musculature of the upper extremities and anterior chest wall, particularly involving the biceps, triceps, deltoids, and pectoralis majors (Fig. 3 *C* and *D*).

MRI of the chest revealed extensive inflammatory changes and enlargement and heterogeneity with scattered foci of hyperintensity through the sternal head of the pectoralis major, visualized deltoid, and biceps brachii muscles, as well as marked dermal thickening and heterogeneity of the subcutaneous tissue of the anterior chest wall (Fig. 3 *E*–*G*).

Noncontrast CT of the pelvis confirmed a stone within the left ureter with associated periureteral stranding, proximal hydroureter, and left‐sided nephrolithiasis, but no hydronephrosis or other abnormalities were seen. Brain MRI was without evidence of masses, hemorrhage, cortical dysplasia or heterotopia, recent infarct, abnormal meningeal enhancement, or enhancing parenchymal lesions. Very mild, nonenhancing, white matter hyperintensities had been reported (Fig. 3 *H* and *I*). Bone mineral densitometry was performed of the hip and spine; the results were normal. The results of a gallium‐68 neuroendocrine PET/CT whole‐body scan indicated there was no definite evidence of suspected oncogenic osteomalacia.

## Discussion

The combination of hypercalcemia with suppressed PTH is suspicious of malignancy. Primary hyperparathyroidism and malignant neoplasms are responsible for more than 90% of all cases of hypercalcemia.^5^, ^6^ Primary hyperparathyroidism was ruled out because of the suppressed PTH level in this patient; therefore, this was a PTH‐independent process. His phosphorus levels were very low and he had a normal PTHrP, ruling out a PTHrP‐mediated malignancy such as non‐Hodgkin lymphoma and solid squamous cell tumors, particularly of the lung and kidney. Also, his SPEP and UPEP were normal. SPEP has a sensitivity ofapproximately 80% for multiple myeloma, and sensitivity is increased to >95% with the addition of serum immunofixation and UPEP/urine immunofixation, which was found to be normal, ruling out multiple myeloma. The results of the whole‐body neuroendocrine PET/CT scan indicated there was no definite evidence of tumor‐induced osteomalacia.

Other causes of hypercalcemia that were ruled out included milk–alkali syndrome caused by anexcessive use of absorbable alkali and calcium, vitamin D intoxication, vitamin A intoxication, sarcoidosis, tuberculosis,  familial forms of primary hyperparathyroidism, Addison disease, some fungal infections,  prolonged immobilization in patients with high skeletal turnover including Paget disease patients, the recovery phase of rhabdomyolysis‐associated acute renal failure, and certain medications including long‐term lithium use resulting in tertiary hyperparathyroidismand hydrochlorothiazide.

The presence of hypercalcemia with suppressed PTH and inappropriately elevated 1,25(OH)_2_D suggests a diagnosis of granulomatous disease. Evaluation for tuberculosis and sarcoidosis at outside institutions was unrevealing.There was no evidence for lymphoma that could also be associated with elevated levels of 1,25(OH)_2_D.

His severe vitamin D deficiency was in part caused by a previous recommendation to eliminate all vitamin D intake because of his granulomas and hypercalcemia.However, his deficiency was exacerbated by the increased circulating levels of 1,25(OH)_2_D and FGF‐23.

1,25(OH)_2_D, when interacting with target tissues, immediately promotes its own destruction by increasing the expression and production of the 25‐hydroxyvitamin D‐24‐hydroxylase (cyp24A1). The elevated blood levels of 1,25(OH)_2_D increase the renal cyp24A1 activity, which not only causes side‐chain cleavage of 1,25(OH)_2_D between carbons 23 and 24 to form the water‐soluble biologically inactive calcitroic acid, but also does the same to 25(OH)D. In addition, FGF‐23 induces the expression of cyp24A1. As a result, there is a marked increase in the catabolism of 25(OH)D, thereby decreasing the blood levels of this metabolite. Therefore, these two mechanisms, coupled with the patient's vitamin D abstinence, accelerated this patient's severe vitamin D deficiency, resulting in extremely low or undetectable circulating levels of 25(OH)D.^7^, ^8^

Despite the improvement of his hypercalcemia, our patient developed extreme fatigue. An outside psychiatric evaluation concluded that, with the improvement in his serum calcium, his inability to get from a sitting to standing position without assistance and his inability to extend his neck to lift his chin from his chest were based on a psychiatric disorder, not a metabolic disorder. In our patient, his extremely low serum 25(OH)D and intermittent low phosphorus levels were contributing factors for his fatigue. Hypophosphatemia is defined as a serum phosphate level <2.5 mg/dL (<0.80 mmol/L) and can be further characterized as mild (approximately 2.0 to 2.5 mg/dL or approximately 0.64 to 0.80 mmol/L), moderate (approximately 1.0 to 2.0 mg/dL or approximately 0.32 to 0.64 mmol/L), or severe (<1.0 mg/dL or <0.32 mmol/L). No specific symptoms suggest hypophosphatemia; rather, symptoms are often nonspecific and depend largely on the cause, duration, and severity. Mild hypophosphatemia is usually asymptomatic regardless of whether it is acute or chronic. Patients may complain of fatigue and weakness; however, whether this is related to the cause or an effect of hypophosphatemia is unclear. Patients with chronic and/or moderate or severe hypophosphatemia are more likely to be symptomatic. Symptoms of severe hypophosphatemia include irritability, confusion, coma, and respiratory difficulty. Prolonged hypophosphatemia with a normal serum calcium or low serum calcium, resulting in an inadequate calcium phosphate product, can also cause osteomalacia, resulting in extended pains in the bones and muscles.

Severe vitamin D deficiency causes proximal muscle weakness. In addition, generalized muscle weakness is the most common symptom of hypophosphatemia; weakness and fatigue are frequent symptoms with acquired hypophosphatemia.^9^ The proximal muscle weakness in our patient with mild hypophosphatemia at the time he presented with severe proximal muscle weakness is likely related to his severe vitamin D deficiency. His alkaline phosphatase was normal. In chronic hypophosphatemia, osteomalacia develops and the alkaline phosphatase is usually in the high or high‐to‐normal range.  We could not find any evidence of osteomalacia in our patient from his medical history or physical examination. This is likely because he had severe hypercalcemia and relatively short‐term and intermittent hypophosphatemia. A normal TMP/GFR in our patient indicates renal conservation of phosphate, and hence a nonrenal cause of his hypophosphatemia.

The sclerosing granulomas (paraffinomas) were diagnosed based on the presence of granulomas (reported in the MRI report), increased muscle bulk, and several nontender indurated areas where silicone/mineral oil was injected based on his physical examination. There are several case reports of 1,25(OH)_2_D‐mediated hypercalcemia from sclerosing granulomas caused by cosmetic injection of silicone and/or mineral oil.^1^, ^10^, ^11^, ^12^ Patients with chronic kidney disease (CKD) and chronic hyperphosphatemia often have elevated circulating levels of FGF‐23. Ahigh level of FGF‐23 is an important predictor of the progression of CKD and a strong predictor of death.^13^ Our patient had mild‐to‐moderate renal failure and intermittent normal and low serum phosphorus levels, which could not explain his elevated FGF‐23 levels. The calculated TRP was normal and the tubular threshold maximum for phosphorus per GFR was low. The low TMP/GFR may be the result of his low serum phosphorus level. A normal TRP and low TMP/GFR in our patient indicate renal conservation of phosphate, and hence a nonrenal cause of hypophosphatemia. The three primary mechanisms of hypophosphatemia include increased renal excretion, decreased intestinal absorption, and shifts from the extracellular to intracellular compartments. The genetic forms of hypophosphatemia are shown in Table 1.

**Table 1:** Genetic Forms of Hypophosphatemia

Autosomal dominant hypophosphatemic rickets
X‐linked hypophosphatemia
Autosomal recessive hypophosphatemia
McCune‐Albright syndrome
Hypophosphatemia with hyperparathyroidism
Hypophosphatemia with renal lithiasis or bone demineralization
Hereditary hypophosphatemic rickets with hypercalciuria

**Figure 4:**
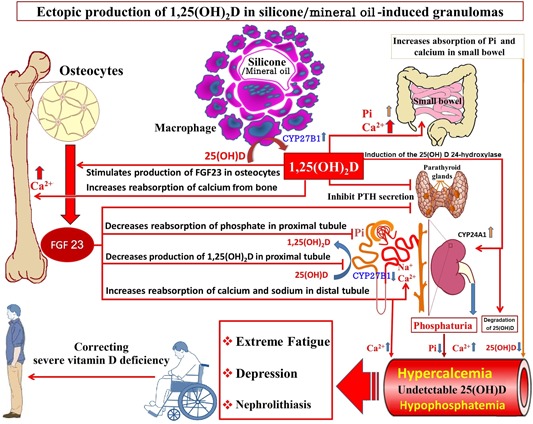
Proposed mechanism of severe fatigue, proximal muscle weakness, hypophosphatemia, and hypercalcemia. The sclerosing granulomas (paraffinomas) were caused by previous self‐administered silicone/mineral oil injections purchased on the Internet. It was advertised as a product that enhances muscle bulk without the use of androgen steroids. His persistently elevated serum 1,25‐dihydroxyvitamin D [1,25(OH)_2_D] levels was from the extrarenal production by the macrophage 25‐hydroxyvitamin D‐1α‐hydroxylase.  The elevated 1,25(OH)_2_D enhances the 25‐hydroxyvitamin D‐24‐hydroxylase (cyp24A1), which increases the destruction of not only 1,25(OH)_2_D, but also 25‐hydroxyvitamin D resulting in severe vitamin D deficiency.  The elevated 1,25(OH)_2_D caused hypercalciuria, hypercalcemia, nephrolithiasis, and renal insufficiency.  The unregulated production of 1,25(OH)_2_D resulted in an increase in the production of FGF‐23 by osteocytes and osteoblasts causing an increase in cyp24A1 activity and hypophosphatemia.  Although the hypophosphatemia was likely a cause for his severe fatigue and muscle weakness, the loss of his proximal muscle function in his shoulders and hips was caused by his severe vitamin D deficiency because the serum phosphate level was normal.  His extreme fatigue and severe proximal muscle weakness markedly improved after receiving small doses of vitamin D (400 IUs daily) and raising his blood level of 25(OH)D from undetectable (<4 ng/mL) to 10 ng/mL.

Phosphate is freely filtered at the glomerulus and then reabsorbed, but proximal tubular reabsorption is inhibited by both PTH and FGF‐23, resulting in hypophosphatemia. In vitro studies in cultured rat calvaria demonstrated that 1,25(OH)_2_D increased FGF‐23.^14^ In addition, it was reported that that FGF‐23 synthesis in rat calvaria was upregulated by 1,25(OH)_2_D.^14^ Therefore, the likely explanation for the markedly elevated blood levels of FGF‐23 in our patient is his elevated 1,25(OH)_2_D level, which in turn was stimulating the osteocytes and osteoblasts to produce this phosphate‐regulating hormone, resulting in hypophosphatemia (Figs. 2, 4).

A trial of corticosteroid therapy has been widely advocated in the acute management of hypercalcemia associated with high levels of 1,25(OH)_2_D, originating from an extrarenal source.^15^ Prior to using antiresorption drugs, all potential causes of osteomalacia had been excluded in this patient. Treatment with systemic steroids, furosemide, calcitonin, ketoconazole, and denosumab resulted in a decrease in serum calcium levels and symptoms associated with his hypercalcemia, but the effects of treatment for his hypophosphatemia and fatigue were limited. With his continued hypercalcemia, there was concern about giving him oral phosphate because this could result in an elevated calcium–phosphate product, producing soft tissue calcification and worsening of his renal failure.However, correcting his severe vitamin D deficiency with small doses of vitamin D_3_ (400 IUs daily) and raising his blood level of 25(OH)D to 10 ng/mL was effective in reversing his severe proximal muscle weakness, allowing him to lift his head, free himselfof his wheelchair, and regain ambulation. This improvement in his proximal muscle function was likely caused by a vitamin D‐dependent effect on skeletal muscle function because his serum phosphate level was normal at this time(Fig. 2). Several studies report an association between vitamin D deficiency and proximal myopathy.^16^, ^17^, ^18^, ^19^ In 30% of patients, it can present as proximal muscle weakness before the biochemical signs of vitamin D deficiency appear, leading to unnecessary investigative workup.^16^, ^17^

A previous study found that proximal muscle strength strikingly improved when 25(OH)D levels increased from <4 ng/mL to 16 ng/mL and continued to improve as the levels increased to >40 ng/mL.^20^ These results are consistent with our observation that a small improvement in 25(OH)D levels from <4 ng/mL to 10 ng/mL dramatically improved proximal muscle strength. A 41‐year‐old male, who presented with hypophosphatemia‐associated osteomalacia caused by a lack of sunlight exposure and adequate vitamin D intake,^21^ had a dramatic improvement in muscle function after receiving vitamin D3 supplementation. He had low serum phosphorus (1.9 mg/dL), with a serum calcium level of 8.1 mg/dL and a 25(OH)D level of 8.1 ng/mL. The patient was thought to have concomitant vitamin D deficiency and possible tumor‐induced osteomalacia. He was given a trial of vitamin D supplementation pending further investigation; in the ensuing 6 weeks he experienced a dramatic improvement in muscle power and regained the ability to climb stairs after 2 months.

Clinicians need to be aware that severe vitamin D deficiency, defined as an extremely low/undetectable 25(OH)D level (ie, at the detection level of the assay, usually >4 ng/mL), can cause severe proximal muscle weakness independent of secondary hyperparathyroidism‐induced hypophosphatemia.^18^, ^19^, ^21^ Furthermore, the persistently elevated blood levels of I,25(OH)_2_D generated by granulomas can indirectly affect phosphate metabolism by increasing the production of FGF‐23, which results in hypophosphatemia.

## Disclosures

All the authors, with the exception of MFH, state that they have no conflicts of interest. MFH is a consultant for Quest Diagnostics, Inc., and Ontometrics, Inc. He is also on the Speakers Bureau for Abbott Laboratories.
